# Advances in the Modulation of Potato Tuber Dormancy and Sprouting

**DOI:** 10.3390/ijms25105078

**Published:** 2024-05-07

**Authors:** Xueni Di, Qiang Wang, Feng Zhang, Haojie Feng, Xiyao Wang, Chengcheng Cai

**Affiliations:** 1 College of Agronomy, Sichuan Agricultural University, Chengdu 611130, China; 2 State Key Laboratory of Crop Gene Exploration and Utilization in Southwest China, Sichuan Agricultural University, Chengdu 611130, China

**Keywords:** potato, dormancy, sprouting, environment factors, carbohydrate metabolism, hormone

## Abstract

The post-harvest phase of potato tuber dormancy and sprouting are essential in determining the economic value. The intricate transition from dormancy to active growth is influenced by multiple factors, including environmental factors, carbohydrate metabolism, and hormonal regulation. Well-established environmental factors such as temperature, humidity, and light play pivotal roles in these processes. However, recent research has expanded our understanding to encompass other novel influences such as magnetic fields, cold plasma treatment, and UV-C irradiation. Hormones like abscisic acid (ABA), gibberellic acid (GA), cytokinins (CK), auxin, and ethylene (ETH) act as crucial messengers, while brassinosteroids (BRs) have emerged as key modulators of potato tuber sprouting. In addition, jasmonates (JAs), strigolactones (SLs), and salicylic acid (SA) also regulate potato dormancy and sprouting. This review article delves into the intricate study of potato dormancy and sprouting, emphasizing the impact of environmental conditions, carbohydrate metabolism, and hormonal regulation. It explores how various environmental factors affect dormancy and sprouting processes. Additionally, it highlights the role of carbohydrates in potato tuber sprouting and the intricate hormonal interplay, particularly the role of BRs. This review underscores the complexity of these interactions and their importance in optimizing potato dormancy and sprouting for agricultural practices.

## 1. Introduction

The potato (*Solanum tuberosum L.*) tuber, a swollen underground stem resulting from the enlargement of the subapical underground stolon, holds a significant position in global agriculture [1]. Ranked after wheat, maize, and rice, potatoes have become a vital staple food crop, taking over the top spot of root and tube crops [2]. Potatoes are therefore considered a cornerstone of global nutritional security and play a vital role in ensuring global food supply and accessibility [3]. This change in status can be attributed to the rich nutritional feature of potatoes, which is characterized by a high starch content, proteins, minerals, essential vitamins, low fat levels, and richness in essential amino acids [3,4]. Grown in more than 100 countries, the potato’s appeal lies in its succulent tuber storage organ, which exhibits numerous uses, from basic food to a major ingredient in industrial food and starch manufacturing [2,5]. Throughout the life cycle of potato tubers, the balance between dormancy and sprouting is pivotal and significantly affects potato growth and yield [6,7]. However, challenges arise in post-harvest scenarios, particularly regarding potato sprouting, which occurs during storage after a break in dormancy. The potato industry faces significant obstacles in preventing unexpected sprouting and the accumulation of sprout growth inhibitors, posing a threat to post-harvest potato safety [8]. Unexpected sprouting during potato dormancy can lead to potato losses during potato storage. Hence, the dormancy period of potatoes should match the storage period to achieve safe storage with bud control, freshness preservation, and loss reduction. On the other hand, inconsistent sprouting in potato seeding can disrupt the uniformity of plant growth, resulting in reduced crop yields, quality, and overall productivity. So, the sprouting time of potatoes should match the sowing period to achieve fast, uniform, and strong seedling emergence. Furthermore, the unpredictability of the potato tuber formation period can lead to early sprouting prior to harvesting. So, it is crucial to align the tuber formation period with the harvesting period to avoid this issue. Therefore, the preservation of potato quality and storability after harvest has become a critical concern for both economic stability and global food security.

During the growth stage of potato tubers, the number of lateral bud (LB) meristems, commonly known as “eyes”, increases and appears as a helical structure on the tuber’s surface [9]. After harvest, these tuber buds predominantly enter a dormant state, resisting sprouting even under conditions that would normally be favorable for such growth [10]. Reust et al. [11] define this dormancy as the interval from tuber onset to the emergence of the initial sprouts, while Emilsson et al. [12] differentiate it between a “rest period”—a phase where the potatoes are intrinsically unable to sprout—and a “dormancy period”, where sprouting does not occur despite optimal conditions. Potato dormancy represents a critical survival strategy, ensuring that tubers sprout only when conditions favor new plant growth. Dormancy is categorized into three distinct types [13] as follows: endodormancy, paradormancy, and ecodormancy. Endodormancy is controlled by internal physiological factors within the tuber itself. During endodormancy, growth inhibitors within the potato prevent the eyes (buds) from sprouting, and the tuber is not responsive to external environmental cues that typically stimulate sprouting. Endodormancy must be broken internally before the tuber can respond to sprouting conditions. Paradormancy is controlled by factors external to the dormant part but internal to the plant. In potatoes, this can be due to the influence of other parts of the plant that are still attached, such as the stolon or the main stem, which may release chemical signals that inhibit the sprouting of the tuber. Ecodormancy occurs when external environmental conditions are not suitable for growth. Unlike internal endodormancy, ecodormancy is caused by external factors such as temperature, light, or nutrient availability. For example, cold soil temperatures or drought conditions can prevent a potato from sprouting even if the tuber itself is not endodormant or paradormant. Each type of dormancy synchronizes the growth of new plants with favorable environmental conditions, thus maximizing the chances of survival and successful growth. The challenge lies in the lack of clear physiological indicators to distinguish these periods. Generally, relative dormancy and environmental dormancy can be reversed by external factors, whereas physiological dormancy is more difficult to change. From a physiological perspective, dormancy in potato tubers begins with the stolon tip’s expansion and ends with the initiation of bud growth. During this stage, various physiological and biochemical events occur; however, these processes do not trigger the morphological changes required for sprouting but affect the number of sprouts produced once dormancy is broken. Both environmental conditions and internal hormonal balance play critical roles during tube dormancy and subsequent sprouting, highlighting the complex dynamic relationship between internal physiology and external stimuli in the post-harvest life of potato tubers [14,15,16].

This review provides an in-depth look at the multifaceted research on potato dormancy and sprouting, with a particular focus on the roles played by environmental factors, carbohydrate metabolism, and hormonal regulation. It investigates the range of environmental influences—from the familiar parameters of temperature, humidity, and light to the less studied effects of magnetic fields, cold plasma, and UV-C irradiation—on the initiation and progression of these key developmental stages. Additionally, it points out the critical function of carbohydrates as the energy source for potato tuber sprouting and dissects the complex network of hormonal interactions, with particular attention given to the role of brassinosteroids (BRs). By synthesizing and evaluating cutting-edge research, this review delivers a comprehensive summary of the contemporary knowledge base and identifies potential pathways for further research. The importance of improving storage conditions, mastering potato dormancy control, and ensuring the durability of harvested potatoes are emphasized. These efforts are crucial for maintaining high levels of agricultural productivity and meeting the food needs of an ever-growing global population. It also highlights the need for ongoing research to explore new methods and technologies that can assist in these efforts.

## 2. Environments

**Figure 1:**
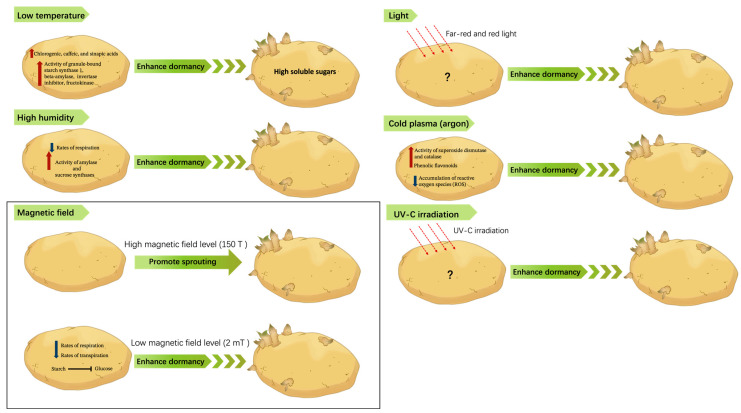
Effects of different environmental and artificial physical factors on potato tuber dormancy.

Potato dormancy is an important post-harvest stage in which the tubers are inactive, preserving their viability until conditions are suitable for growth. The transition from dormancy to sprouting is marked by profound structural and biochemical transformations within the tuber [15,17]. The interplay of genetic factors and environmental stimuli, including light, temperature, humidity, water availability, and nutrients, is pivotal in coordinating the end of dormancy and subsequent sprouting and flowering (Figure 1) [18,19]. Understanding this delicate balance is crucial for the effective management of potato crops, ensuring timely sprouting and optimal yield.

### 2.1. Temperature

Temperature is a fundamental determinant in the management of potato tuber dormancy, as it directly affects numerous physiological and biochemical processes occurring within tubers. Low temperatures can effectively extend the dormancy period, thus improving storage stability [10,20]. In contrast, elevated storage temperatures can accelerate the physiological aging of potato tubers, consequently shortening the dormancy period [6]. Typically, within a storage temperature range of 3–20 °C, tuber dormancy duration is inversely related to temperature [10]. Persistent exposure to temperatures of 2 °C or below, and 30 °C or above, disrupts potato tuber dormancy, while sprouting begins once temperatures stabilize within a moderate range [21].

Studies have indicated that the respiration rate of potato tubers reaches a minimum at around 4 °C and subsequently increases with temperatures rise [22]. Among the polyphenols in potatoes, phenolic acid and flavonoid are particularly abundant, with chlorogenic and caffeic acids being the most prominent phenolic acids [23,24,25]. Notably, increased levels of chlorogenic, caffeic, and sinapic acids were observed after 90 days of storing tubers at 3 °C. It is evident that storage temperature has a significant impact on tuber metabolism and the levels of antioxidant compounds, which enhances their antioxidant capacity [26]. A study utilizing both transcriptomic and proteomic methods provides insights into the mechanisms of adaption of potato tubers to cold storage (as cultivars, DSP 186 and 287, and nine Kufri versions). The research revealed that soluble sugars, including sucrose, glucose, and fructose, increase significantly because of the enzymatic activities of granule-bound starch synthase 1 (responsible for elongating the amylose chains in starch granules), beta-amylase (BAM, catalyzes the hydrolysis of the non-reducing ends of α-1,4 glycosidic bonds in starch chains, resulting in the production of maltose), sucrase inhibitor (regulates the activity of sucrase enzyme), and fructokinase (catalyzes the phosphorylation of fructose) at low temperatures. Meanwhile, the study identified the induction of fifteen heat shock proteins, which are vital for protecting and repairing cell damage under cold stress [27].

Warm and cold environmental conditions during the growth stage of potato tubers significantly influence their subsequent dormancy length. Warm conditions during the developmental period typically result in a shortened post-harvest dormancy period. In contrast, tubers that mature in cooler climates often benefit from a longer dormancy period. Thus, warmer growing seasons are frequently correlated with shorter dormancy duration, while cooler years tend to be associated with longer tuber dormancy [28]. Remarkably, different potato varieties awaken from dormancy at different intervals despite being stored under uniform cold conditions. This variation demonstrates genetic differences among cultivars and their unique dormancy mechanisms. After analyzing the gene expression differences among eight potato cultivars, including three breeding lines derived from *S. brevicaule* and *S. tuberosum* (15329-03, 15323-04, and 15328-26), as well as cultivars such as ‘Doremi’, ‘Lady Claire’, ‘Summer’, ‘breeding line 575.04.01’, and ‘breeding line Crop 20’, it became evident that only specific subset of genes within these cultivars exhibited consistent expression patterns [29]. This variation illustrates the genetic diversity of cultivars, suggesting that each cultivar has its own unique dormancy management parameters. Such insights are critical for breeding programs aimed at enhancing cold storage tolerance and extending the shelf-life of different potato varieties. Understanding these genetic underpinnings can improve post-harvest management tailored to the specific physiological needs of each variety.

### 2.2. Humidity

During potato dormancy, water evaporates from the tuber surface into the surrounding air, with the rate of loss being proportional to the difference in vapor pressure between the intercellular space and ambient humidity. Water loss during storage not only reduces the quality of potatoes but also results in significant weight loss, thus diminishing their overall value. High humidity is crucial for optimal wound healing and is essential to minimize weight loss during the dormant phase of storage [30]. Maintaining a high relative humidity close to 100% is anticipated to minimize total mass loss during potato storage. Investigations have been conducted to confirm this hypothesis since the 1970s [31]. During the dormancy-breaking stage of potato tubers, a relative humidity of approximately 85–90% is considered optimal [15,32]. Conversely, at relative humidity levels ranging from 38% to 66%, tubers commence sprouting, resulting in significant weight loss as sprouting persists over time [33]. Humid conditions decrease oxygen access in the tubers, causing an energy shortfall that is offset by a shift toward glycolysis and fermentation for energy production [34]. This condition can be further controlled by reducing secondary metabolism and breaking down starches and storing proteins to reuse sugars and amino acids. Such conditions may also elevate the levels of Tuber Storage Protein (TPS) and potentially ABA, which could extend potato dormancy [34]. Nonetheless, an increase in rainfall and a higher sielianinov coefficient during the vegetation season have been correlated with a longer dormancy period, suggesting that more subtle climatic patterns may influence the dormancy lifecycle [35].

### 2.3. Light

It has been discerned that diffused light can slow down the physiological aging process of seed tubers, suggesting that both light and darkness are pivotal environmental factors affecting the dormancy and sprouting stages of potato development [32]. In general, plant sprouts tend to develop in dark conditions, with signals from the seed’s cell wall promoting the growth of elongated, yellowish sprouts. This contrasts with the green sprouts that are produced when potato tubers sprout in the presence of light [15]. When harvested potatoes are stored under conditions of low-intensity daylight, the sprout growth rate is impacted. Notably, sprouted tubers kept at temperatures of 10 °C or 18 °C exhibit halted sprout growth when moved from darkness to fluorescent light. On the other hand, tubers moved from light to dark conditions experience a resurgence in sprout growth, progressing at a rate similar to those that have been kept in continuous dark conditions [36]. This indicates that light exposure significantly influences sprout development of potato tubers during post-harvest storage [37].

Recent research has lightened the significant relationship between the light environment surrounding the mother plant and potato tuber dormancy. Studies indicate that exposure to 460 nm blue light can shorten the dormancy period of microtubers of the ‘Favorita’ cultivar. Additionally, a notable inverse relationship was found between the levels of sucrose and starch in microtubers and their dormancy period. This suggests that the application of white light can prolong the dormancy period by reducing the concentration of these two carbohydrates [38]. Moreover, potato tubers of the ‘Asterix’, ‘Folva’, and ‘Mandel’ cultivars treated with red LED light at 660 nm proved most effective in suppressing sprout growth even at very low light levels (10–100 nmol m^−2^ s^−1^), whereas far-red LED light at 735 nm was more effective at higher light intensities. These findings provide valuable insights into the manipulation of light to control potato sprout development and dormancy [39].

### 2.4. Other Environmental Factors

Weak magnetic fields (WMFs) have been shown to potentially exert adverse effects on the sprouting and early growth of seedlings across various plant species [40]. This suppression of growth is attributed to a reduction in proliferative activity within the root meristems, which includes an obstruction of cell division, overall growth processes, and cell reproduction cycles. Furthermore, WMFs appear to influence metabolic functions, with notable impacts on enzymatic activity and calcium homeostasis within plant cells. These findings suggest that even low-intensity magnetic fields can have profound effects on plant development at the cellular and biochemical levels.

Innovative research on the application of magnetic fields (MFs) has revealed promising methods to enhance seed sprouting and increase agricultural yields [41]. Research on vegetative growth and the sprouting process of dormant potato tubers indicates that exposure to a magnetic field can significantly accelerate sprouting. One specific study revealed that when tubers of the ‘Banba’ and ‘Necta’ cultivars were subjected to a magnetic field with a strength of 150 millitesla (mT) for a duration of 72 h, the sprouts appeared significantly faster. Sprouting occurred after only 17 days for ‘Banba’ and after just 14 days for ‘Necta’, compared with the 31.8 and 39.5 days observed in the respective controls. This suggests that magnetic treatment could reduce sprouting time by nearly half when compared with the non-treated control [41]. The method also reflects findings in soybean seeds, which exhibited improved sprouting rates when treated with 150 and 200 mT magnetic fields [42].

However, exposing potatoes of the ‘Shangi’ cultivar to lower strengths of direct current (DC) magnetic fields (0.5, 1.0, and 2.0 mT) for 60 s or alternating current (AC) magnetic fields (0.4, 0.6, and 0.8 mT) for 80 s resulted in fewer sprouts per tuber [43]. Additionally, these MF-treated potatoes showed less weight loss during the same storage period compared with untreated controls. Singh et al.’s [40] research proposes that magnetic fields can reduce the respiration and transpiration rates in stored tubers, thereby minimizing weight loss. It is suggested that the MF may suppress the conversion of starch to glucose, thus reducing the respiration rate, which in turn decreases water loss and extends dormancy [40]. Further experiments using different magnetic strengths (1, 2, and 3 mT) and exposure times (20, 40, and 80 s) successfully prolonged the shelf life of the ‘Shangi’ potato variety by approximately one month.

The varying effects of magnetic fields on potato dormancy and sprouting indicate that the energy level of the MF is a key factor; higher levels promote sprouting, while lower levels may enhance dormancy (Figure 1). To fully exploit the potential of magnetic field treatments in agriculture, more in-depth research is required to understand the underlying mechanisms.

Cold plasma, also known as non-thermodynamic equilibrium plasma, is a novel non-thermal technology that applies energy to a gas, causing it to emit various active species such as free radicals, electrons, ions, and ultraviolet light [44]. Since the electron temperature is very high and the ion and neutral gas temperatures are close to room temperature, cold plasma is very suitable for use in chemical processes and temperature-sensitive items such as crops [45,46]. Research has shown that 20-s argon (Ar) plasma jet treatments can enhance the antioxidant system of potato tubers by affecting enzymes, such as catalase, peroxidase, and superoxide dismutase, and increasing total phenolic flavonoids, which helps in controlling reactive oxygen species accumulation. This increases the antioxidant capacity of the tuber and inhibits early sprouting. On the other hand, a 40-s treatment damages the bud eye tissue of the ‘Xisen no. 6’ cultivar, preventing sprouting altogether. These findings demonstrate the potential of cold plasma treatment in agricultural applications, particularly in managing sprouting and extending potato dormancy [47].

UV-C irradiation has been identified as an effective method for preserving potatoes by reducing sprouting and weight loss during storage [48]. UV-C-treated potatoes exhibit very low levels of rot and can be stored for over eight months without signs of shrinkage [49]. Complementary findings revealed that a low dose of 50 Gy irradiation applied 10 days after harvest is effective in arresting sprout growth in ‘Agria’ cultivar potato tubers stored at 8 °C [50]. The inhibition of sprouting at 16 °C required a higher dose of 150 Gy, but this higher dose also led to a significant reduction in ascorbic acid levels, indicating that although irradiation is efficient in sprout control, it may induce various metabolic changes. The precise molecular mechanisms triggered by UV-C irradiation remain to be fully understood, and further research is needed to assess its effects and to elucidate the molecular alterations that occur. Nonetheless, UV-C treatment has been shown to enhance water retention within the cells, thereby delaying sprouting and reducing weight loss during the storage of potato tubers [51].

## 3. Carbohydrate Metabolism

Potato tubers transition from a sink in the vegetative phase to a source in the vegetative reproductive phase, with their dormancy and sprouting cycles tightly coordinated with environmental factors to achieve optimal plant fitness and yield. The tubers’ metabolic state is pivotal in determining their developmental outcomes. As sugars are key components in these storage organs, their management is integral to the regulation of the dormancy-to-activity transition in geophytes. Carbon sources, particularly sugars, are essential for bud growth post-dormancy, with the plant finely tuning sugar metabolism to balance between dormancy and sprouting. Starch, mainly composed of amylose and amylopectin, is the principal storage polysaccharide in green plants and is abundant in nature. The behavior of starch during tuber dormancy is a critical aspect of potato physiology. During the dormancy phase, starch serves as a stable form of energy storage that the plant can then use to support sprouting and subsequent growth. When a potato enters dormancy, metabolic processes slow down, and starch remains relatively inert. However, as dormancy progresses and eventually ends, enzymes like amylases and phosphorylases become active, breaking down starch into simpler sugars. These sugars are then available to support the growth of sprouts as the potato transitions from a resting state to an active growth state [52].

Throughout the dormancy period, the starch in potato tubers remains metabolically and structurally unstable, suggesting that tubers undergo ongoing biochemical and physical changes despite their dormant state. Studies have shown that the average size of starch grains in stored potato tubers increases, with a concomitant decline in the frequency of smaller grains [53]. This observation suggests that starch granules, especially the smaller ones, may undergo a fusion process during tuber dormancy. The typical feature of potato and yam starch is that the amylopectin double chains are arranged in a hexagonal shape, indicating a B-type polymorphic structure [54,55]. The stability of the starch structures, inferred from melting temperatures, is compromised during the dormancy period, as shown by a decrease in melting thermodynamic parameters [56,57]. Thus, dormancy appears to be associated with an increase in structural defects within the starch matrix. The storage stage not only alters the starch’s crystalline structure but also leads to the proliferation of these defects [53]. Notably, the melting of starch at lower temperatures suggests the dissolution of more defective crystalline lamellae relative to those that melt at elevated temperatures. These defective regions—amorphous and disordered—are preferential sites for enzymatic hydrolysis [58]. It is posited that these disordered starch structures, which melt during dormancy, especially at lower temperatures, are primed to initiate starch hydrolysis, a crucial step in the initiation of tuber sprouting.

**Figure 2:**
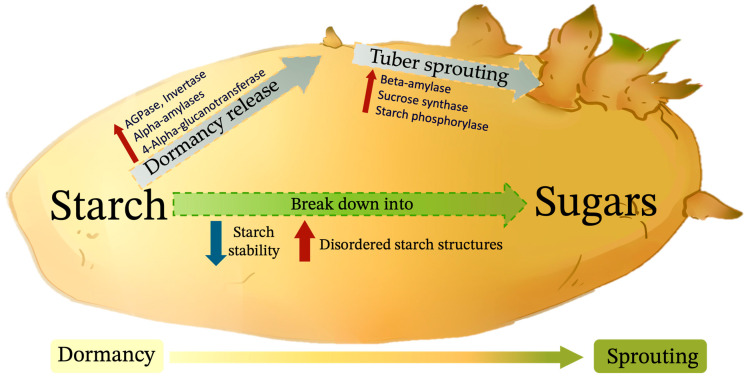
Carbohydrate metabolism occurs during potato dormancy and sprouting. The gradual transition from the dormant tuber stage to the sprouting stage is shown from left to right.

During potato dormancy, starch is broken down into sugars (Figure 2), especially in cooler conditions [48]. The saccharification of starch in potatoes is likely related to the enhanced activity of sucrose synthase enzymes throughout storage, indicating a link between starch saccharification and the end of dormancy (the onset of sprouting) [59]. Transcriptome analysis of the potato cultivar ‘Longshu 3’ during the release of tuber dormancy revealed a high expression of genes involved in starch and sucrose metabolism such as *ADP-GLUCOSE PYROPHOSPHORYLASE* (AGPase, catalyzing the formation of adenosine diphosphate glucose from glucose-1-phosphate), *ALPHA -AMYLASES* (cleaving the α-1,4 glycosidic bonds in the starch chain to break down starch into smaller sugar units), *4-ALPHA-GLUCANOTRANSFERASE* (catalyzing the transfer reaction of glycosyl groups within starch molecules, leading to the rearrangement of starch), *FRUCTOKINASE* (catalyzing the conversion of fructose into 1-phosphofructose), *INVERTASE* (breaking down sucrose into glucose and fructose), and *SUCROSE SYNTHASE* (a key enzyme in the sucrose metabolism process in plants, serving as a glycosyltransferase), with a shift to genes aiding sucrose provision and starch breakdown during dormancy release. Following the emergence of buds (tuber sprouting), there is a notable increase in the gene expression of *SUCROSE SYNTHASE*, *BAM*, and *STARCH PHOSPHORYLASE* (an important enzyme involved in the starch metabolism process) [60]. Although changes in AGPase and starch phosphorylase activity precede visible sprouting, the downregulation of some carbohydrate degradation genes suggests that release from dormancy is not dependent on extensive reserve use. Sucrose metabolism necessitates the presence of inorganic pyrophosphate (PPi); thus, the removal of cytosolic PPi would inhibit sucrose breakdown [16]. Inorganic pyrophosphatase (PPase) catalyzes the hydrolysis of PPi into two orthophosphate molecules. *PPase* overexpression in the potato cultivar ‘Gannongshu 2’ results in increased soluble sugars and earlier sprouting, whereas *ppase*-RNAi leads to lower sugar content and delayed sprouting [61]. It is worth noting that the timing and rate of starch conversion are influenced by multiple factors, including temperature, hormonal changes, and the physiological state of the tuber.

While environmental factors are known to influence carbohydrate levels, the detailed mechanisms of how factors such as oxygen levels and humidity interact with sugar and starch metabolism to affect dormancy are not fully understood. A better understanding of how environmental factors affect carbohydrate metabolism could lead to more precise storage conditions that extend the dormancy period without the use of chemical sprouting inhibitors.

## 4. Hormones

**Figure 3:**
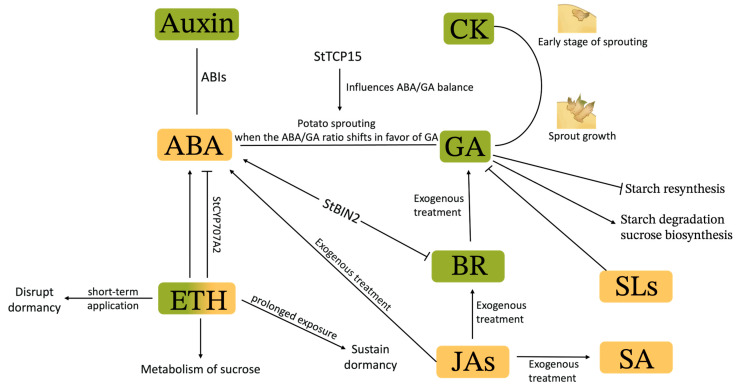
The relationships among hormones during potato dormancy and sprouting. Hormones promoting potato sprouting are labeled with green, those prolonging potato dormancy are labeled with yellow, and hormones exhibiting dual effects are labeled with green and yellow gradations.

The coordination of potato tuber dormancy and sprouting is a delicate process controlled by complex interactions of hormones. These naturally occurring compounds are pivotal at various junctures of the dormancy-to-sprouting continuum, ensuring the seamless transition crucial for potato development [15]. As regulators, hormones like abscisic acid (ABA), gibberellins (GA), cytokinins (CKs), auxins, ethylene (ETH), brassinosteroids (BRs), jasmonates (JAs), strigolactones (SLs), and salicylic acid (SA) each play distinct roles (Figure 3). Understanding the dynamic balance and regulation of these hormones is critical to the scientific exploration of plant biology and the practical management of crop production and post-harvest storage.

### 4.1. Abscisic Acid

Abscisic acid (ABA) is a key phytohormone known for its role as a growth inhibitor in plants and plays a vital role in the dormancy of potato tubers. In potato tubers, ABA was initially discovered within inhibitor complexes that encompassed other acidic growth inhibitors, including para- and ortho-coumaric acids, as well as cinnamic and salicylic acid derivatives. At harvest, tubers typically exhibit high levels of ABA, which gradually decreases during storage—a change that corresponds to the breaking of dormancy. Specifically, ABA concentrations rise in both the eyes and the parenchyma tissues at the onset of dormancy, peaking during deep dormancy, and then significantly decline as the dormancy period ends [62]. The transition from dormancy to active growth is marked by the degradation of ABA, tipping the balance toward the actions of other growth-promoting hormones, such as GA [63]. The interaction between ABA and GA is subtle; as noted by researchers Krauss and Marschner [64], ABA can inhibit the effects of GA and effectively play a balancing role. This inhibition is a defense mechanism that protects the tuber from cold damage by halting DNA and RNA synthesis, thus maintaining the cells in the G1 phase of the cell cycle. It is only when the ratio of GA to ABA is tilted toward GA that the potato cell escapes this protective state. As GA levels rise relative to ABA, cell division is promoted, leading to the sprouting process. This relationship between ABA and GA is the heart of the physiological regulation of potato dormancy and sprouting, and understanding this hormonal dialogue is crucial for optimizing agricultural practices and improving potato crop management [65]. *StTCP15* has been observed to modulate the dynamic equilibrium between ABA and GA. The overexpression of *StTCP15* in the potato cultivar ‘Eshu 10’ caused a marked decrease in the ABA/GA ratio, which promoted the sprouting of potato tubers. In contrast, lines with downregulated expression of the *StTCP15* gene exhibited delayed sprouting, associated with an increased ABA/GA ratio [66].

The biosynthesis of cellular ABA predominantly takes place within chloroplasts and the cytoplasm. Initially, zeaxanthin epoxidase (ZEP) catalyzes the conversion of the carotenoid zeaxanthin within the chloroplast into all-trans violaxanthin. Subsequently, the intermediate compound violaxanthin undergoes catalysis by the enzyme nine-cis epoxycarotenoid dioxygenase (NCED) to form xanthoxin within the chloroplast. This xanthoxin is then transported to the cytoplasm. Once in the cytoplasm, it is transformed into abscisic aldehyde, which is further oxidized by the enzymes short-chain dehydrogenase/reductase (SDR) and abscisic aldehyde oxidase to yield the biologically active form of ABA [67]. Members of the cytochrome P450 monooxygenase (CYP707A) superfamily catalyze the conversion of bioactive ABA into hydroxy ABA, a process that deactivates ABA [68]. Consequently, the pivotal enzymes responsible for ABA synthesis include ZEP, NCED, short-chain dehydrogenase/reductase, and abscisic aldehyde oxidase, all of which positively contribute to ABA metabolism. In contrast, members of the cytochrome P450 monooxygenase (CYP707A) superfamily play a negative regulatory role in ABA metabolism, facilitating the deactivation of ABA through their catalytic action [69]. Within the cortical tissue, it was found that the expression patterns of *StNCED1* and *StNCED2*, genes integral to the synthesis and regulation of ABA, exhibited distinct differences. Specifically, the expression level of *StNCED1* reflected the concentration of ABA, indicating a strong correlation between the two. Conversely, the abundance of *StNCED2* transcripts peaked at the beginning of the storage period, indicating dormancy onset, and subsequently underwent significant fluctuations. It decreased significantly between day 48 and 82, rose again at day 97, and ultimately fell to its lowest level. In view of these observations, the precise roles of *StNCED1* and *StNCED2* in modulating tuber dormancy and ABA metabolism remain incompletely understood [70]. The dynamic balance of ABA accumulation moves toward degradation, and potato dormancy is released. Research by Liu et al. [71] underscored this balance by demonstrating that overexpression of *StCYP707A* in the potato cultivar of ‘Désirée’, which corresponds to enhanced ABA catabolism, leads to decreased ABA levels, and increased GA levels, hence accelerating the release of dormancy. Moreover, the interaction between ABA with other signaling molecules like nitrogen oxide (NO) and hydrogen peroxide (H_2_O_2_) also plays a significant role in dormancy dynamics. NO, in particular, promotes the expression of *StCYP707A1*, which is involved in ABA breakdown, and inhibits *StNCED1*, thus tilting the hormonal balance toward dormancy release and tuber sprouting [72]. The possible connection between ABA metabolism and nicotinamide adenine dinucleotide (NAD+) signaling is an exciting frontier in plant science. Proteins within the NAD+ biosynthetic pathway may influence seed germination and the ABA-mediated control of sprouting, suggesting that the network of regulatory mechanisms controlling plant development is more extensive and interconnected than previously understood [73].

Despite the well-established effects of ABA on inhibiting sprouting and maintaining tuber dormancy, there are still significant shortcomings in its action. Researchers have yet to fully characterize the interaction between ABA and other plant hormones or how these dynamics are modulated by genetic and environmental factors. By deciphering the complexities of ABA signaling pathways and their integration with plant developmental factors, we may enhance our ability to manipulate dormancy and sprouting processes.

### 4.2. Gibberellin

Gibberellins (GAs) facilitate the termination of dormancy by stimulating the synthesis of DNA and RNA and by abbreviating the G1 and S phases in the cell cycle [74]. GA acts as an antagonist to ABA in the regulation of seed germination, as evidenced by GA-deficient mutants that exhibit elevated levels of ABA. The analysis of GA1, GA19, and GA20 concentrations in potato tubers reveals that the highest levels of gibberellins correlate with vigorous sprout growth. While the administration of GA19 does not markedly alter the duration of tuber dormancy, it does promote the growth of sprouts in tubers that are no longer dormant. Notably, although GA1 and GA20 are present at high concentrations, the levels of GA19 show a substantial increase during advanced storage periods, coinciding with the initiation of shoot growth [75]. Exogenous application of GA, GA1, and GA20 accelerates the sprouting of seed potatoes and ensures vegetative growth, while GA12, GA19, and GA8 are inactive during dormancy release [76]. These observations suggest that endogenous GAs play a role in regulating sprout growth after the termination of dormancy but not in the forward process of tuber dormancy.

GA impedes starch resynthesis through the downregulation of *AGPase* and *GRANULE-BOUND STARCH SYNTHAS* (involved in the enzymatic reactions of starch synthesis) genes while enhancing the expression of *BAM* and *UDP-GLUCOSE PYROPHOSPHORYLASE* (involved in the biosynthetic pathway of sucrose), thereby counteracting the suppressive impact of INH1/2 on invertase activity, both acid (AI) and neutral (NI) invertases. This action facilitates starch breakdown and the synthesis of sucrose [77]. The biosynthesis and catabolism of GAs involve several steps and enzymes. Non-bioactive GAs serve as precursors and intermediates, which are then converted into bioactive forms mainly by GA 20-oxidases (GA20ox) and GA 3-oxidases (GA3ox). On the other hand, GA 2-oxidases (GA2ox) or other P450 monooxygenases, such as the elongated uppermost internode (EUI) in *Arabidopsis* or rice, deactivate bioactive GAs to non-bioactive forms [78]. Thus, GA20ox and GA3ox enzymes play critical roles as promoters of bioactive GA synthesis, thereby facilitating GA metabolism. Conversely, GA2ox and EUI enzymes are instrumental in the breakdown of bioactive GAs, thus serving as repressors of GA activity. Transgenic tubers (cultivar of ‘Solara’) expressing *GA2ox* throughout the organism presented not only a significantly shorter dormancy period but also developed longer and thinner sprouts compared with control tubers [79]. This shortening of dormancy was significantly more pronounced in clones with constitutive expression than in those with leaf-specific overexpression, indicating that GAs synthesized locally within the tuber are more effective in dormancy regulation than those transported from the foliage [79]. Furthermore, transgenic cloning experiments revealed reduced expression of the *StGA20oX1* gene, resulting in decreased levels of the gibberellin GA1, leading to the formation of shorter sprouts. Interestingly, these changes in gene expression and gibberellin levels did not affect the length of the dormancy period. This observation highlights the intricate effects of gibberellins on the process of sprout development [80,81]. GA signaling, typically obstructed by DELLA proteins during dormancy, is reactivated at the end of dormancy through the interaction between GA and the Ga-insensitive DWARF 1 (GID1) receptor. This binding event drives the degradation of DELLA proteins, augmenting GA accumulation and facilitating the emergence of sprout growth [78]. It has been observed that artificial elevation in GA levels through external application or expression of GA biosynthetic genes promotes untimely sprouting in tubers. Notably, the surge in intrinsic levels of GAs coincides with the onset of sprout growth, implying that GAs mainly enhance the elongation of sprouts that have already exited dormancy [75].

Current research on GA has elucidated its significance in the regulation of potato dormancy and sprouting, yet there is still a lack of understanding. The complexity of the signal transduction pathways involved in GA throughout dormancy and the subsequent sprouting stage requires further exploration. An integrated approach that considers the interactions among hormonal signals, genetic regulation, and environmental stimuli could illuminate a holistic model of how GAs control dormancy. Delving deeper into the working mechanisms of GA could not only enhance understanding of potato biology but may also lead to advances in agriculture. With these insights, it will be possible to regulate the uniformity of potato sprouting consistently and control the timing of dormancy release precisely, thereby optimizing the crop management strategies.

### 4.3. Cytokinins

Cytokinins (CKs) are crucial plant hormones that, among other functions, regulate the G1-to-S phase transition within the cell cycle by inducing the expression of *CYCLIN D3* genes [82]. These hormones, particularly kinetin and benzyladenine (BA), have also been shown to stimulate tuber formation in vitro when administrated with higher sucrose concentrations and in complete darkness, suggesting that they have significant effects on plant morphogenesis [83]. CKs, vital plant hormones derived from adenine, have two natural variants, including isoprenoid and aromatic, which are differentiated by their respective side chains at the N6 position, with isoprenoid CKs having an isoprenoid side chain and aromatic CKs featuring an aromatic group. The isoprenoid cytokinins encompass several types, including N6-isopentenyladenine (iP), trans-zeatin (tZ), cis-zeatin (cZ), and dihydrozeatin (DZ) [84].

Previous research has revealed the pivotal role of cytokinins in potato tuber sprouting. The application of exogenous cytokinin to dormant tubers has been shown to shorten the dormancy phase, triggering earlier sprouting events. This external stimulus appears to be consistent with changes in endogenous hormones within the tuber itself, where the concentrations of isopentenyl adenine (iP) and trans-zeatin (naturally occurring cytokinins) increase before the sprouts start to grow. These findings underscore the intrinsic role of cytokinin in the regulation of potato tuber development. The observed increase in the levels of iP and tZ prior to the onset of sprouting indicates that these hormones may serve as endogenous signals for the tuber to exit dormancy and initiate growth. The rise in their levels highlights the readiness of the tuber to initiate the development of sprouts. Research has highlighted cis-CK as a potential regulator of potato tuber dormancy [85]. Evidence pointed out the significance of cytokinin homeostasis in the sprouting process, underscored by the increased expression levels of *CISZOG* and *UGT85A1* involved in CK metabolism. The role of cytochrome P450 monooxygenases, particularly CYP735A, in CK biosynthesis has also received attention. CYP735A catalyzes the hydroxylation of isopentenyladenosine diphosphate (iPRDP) and isopentenyl adenosine triphosphate (iPRTP) nucleotides, yielding trans-CK in the “Zeatin biosynthesis” pathway [86,87]. The active expression of *CYP735A* in sprouting tuber buds is consistent with the upregulation of CK biosynthetic signaling during sprouting [88].

CK is involved in the early stage of sprouting, which induces the termination of dormancy, while GA causes sprout growth [89]. CKs are essential at the onset of sprouting, setting the stage for dormancy release; however, it is the action of GAs that drives the subsequent sprout growth. Thus, without GA, sprouts will not elongate further. The interaction between CK and GA underscores the delicate hormonal equilibrium necessary for the normal development of potato dormancy and initiation of sprouting. Hartmann et al. [79] conducted pioneering work to manipulate cytokinin levels in transgenic potatoes (cultivars of ‘Solara’) through the expression of *ISOPENTENYL TRANSFERASE* and *CYTOKININ OXIDASE/DEHYDROGENASE* genes. Their work illustrated the dynamic interaction between CK and GA during the stages of dormancy and sprouting, and a synergistic effect was observed in accelerating sprouting. These insights underscore the likelihood that genes involved in regulating hormone synthesis play a pivotal role in influencing dormancy and sprouting processes [6]. The manipulation of CK levels was further explored by Zubko et al. [90], in which the impact of the *Sho* gene from Petunia hybrida was assessed, which is a gene involved in the regulation of cytokinin synthesis during potato organ growth. Expression of the *Sho* gene in transgenic potatoes (cultivars of ‘Désirée’) was associated with a significantly reduced dormancy period. Seeds harvested from these modified potatoes not only sprouted faster but also exhibited enhanced shoot and stolon growth. RNA sequencing (RNA-seq) analyses indicated complex regulatory dynamics, with a general downregulation of cytokinin-activated signaling juxtaposed with an upregulation of the “Zeatin biosynthesis” pathway, as evidenced by increased expression of *CYP735A*, *CISZOG*, and *UGT85A1*. This suggests a shift from cytokinin utilization to its synthesis during sprouting [91]. Potato tuber sprouting is closely related to the action of CKs, indicating that the signaling and balance of CKs are essential in facilitating sprouting initiated by GAs. This emphasizes the importance of CK dynamics for the successful emergence of new sprouts.

Indeed, while recent research studies have provided some descriptive analyses regarding fluctuations in cytokinin levels throughout various development stages of the potato, these studies generally do not delve into the detailed mechanisms that might explain how cytokinins control dormancy release or maintenance processes.

### 4.4. Auxin

Auxin, like GA and CK, plays a pivotal role as a growth-promoting agent in regulating tuber dormancy and subsequent sprout development [15]. Unique among plant hormones, auxin possesses a special system for long-distance transport, influencing every aspect of plant development, from embryogenesis to the spatial configuration of plant architectures. Although several natural and synthetic auxins have been recognized, indole-3-acetic acid (IAA) represents the most prevalent auxin form in plants [92].

The dynamic regulation of auxin levels is particularly evident in the metabolism of mature tubers, with its concentrations varying at different developmental stages—initially higher at the stolon tip during the early stages of tuber formation and decreasing as the tuber matures [92]. In a detailed study of the role of IAA in potato tubers, researchers assessed the concentrations of both free and conjugated IAA in different tuber regions (eyes, sub-eye tissues, and pith) from the onset of dormancy to the initiation of sprouting at storage temperatures of 3 °C and 23 °C. The findings displayed that IAA levels were highest in the eyes, with a significant rise in free IAA observed from the point of harvest to the end of dormancy, independent of storage temperature. At 23 °C, conjugated IAA was more abundant in the eyes and sub-eye regions as compared with free IAA, whereas at 3 °C, elevated IAA levels were confined to the eyes [6]. The fundamental role of auxin in bud differentiation and its stimulatory effect on bud growth are well documented. Studies have established a positive relationship between auxin levels and the termination of tuber dormancy [93,94].

The regulation of seed germination by auxin is mediated through the Auxin Response Factor (ARF). Faivre-Rampant et al. [95] proposed the *ARF* gene as a potential indicator of meristem reactivation in potato tubers, noting that *ARF1* gene expression was significantly upregulated concurrently with tuber dormancy termination, which may be intricately related to the processes of dormancy and sprouting [96]. The transition from a state of dormancy to active seed growth is orchestrated by complex interactions between auxin and ABA signaling pathways that are intricate in plant physiological processes. Studies have unveiled that auxin and ABA do not act alone but regulate seed dormancy in a synergistic manner [97]. As mentioned before, ABA, well-known for its germination-inhibitory properties, exerts its effects predominantly through a group of proteins known as ABIs (ABA-insensitive proteins). Liu et al. [98] provided evidence that two auxin response factors, ARF10 and ARF16, act as transcriptional activators of ABI3. Data from their published work elucidates the necessity of *ARF10* and *ARF16* in maintaining *ABI3* expression levels, which are crucial for the proper regulation of seed dormancy and germination. Further investigations of the interrelationship among these hormones revealed that mutations within the *abi3* gene reduce the effectiveness of both auxin and ABA on seed germination in *Arabidopsis*, signifying a critical role for ABI3 in the hormonal control mechanism. Based on this knowledge, recent studies have identified ABI3, as well as ABI4 and ABI5, as core elements that drive the transition from dormancy to sprouting [99]. Delving deeper into the specific aspects of potato tuber dormancy, the homologous gene *StABI5* has been identified as a significant regulator of tuber dormancy. *StABI5* appears to control tuber dormancy by regulating the auxin signaling pathway. This regulation extends to StABI5’s influences on downstream genes, including *SUAR* and *AUX*, which are part of the auxin-signaling cascade [100].

The innovative application of the *TMS1* gene (involved in auxin synthesis) in transgenic plants has achieved remarkable outcomes in enhancing auxin biosynthesis, which in turn significantly increases tuber yield and suppresses undesirable sprouting. One of the most striking findings in these transgenic potato specimens is the interaction between auxin and CK, where CK appears to elevate the expression of auxin receptor genes, thereby amplifying the auxin signal transduction pathway [92]. Another noteworthy observation in this field is the involvement of the hydrophobic loop of the auxin transporter StPIN4. Relevant research indicates this loop is subject to phosphorylation by the protein kinase StCDPK1, an event that occurs before the sprouting of tubers, suggesting deeper post-translational control of the auxin transport mechanism [101].

Law and Suttle’s study [75] raised an interesting paradox regarding the application of exogenous auxins such as IAA and NAA. After tuber dormancy was broken, the application of these auxins was found to promote sprout growth, an effect commonly associated with auxin’s promotion of cell elongation. However, it was also established that high concentrations of auxin actually inhibited this process. The delicate balance in auxin concentration determines diametrically opposed biological responses, underscoring the complexity of hormonal regulation in plant development.

Although auxins are recognized as being involved in plant development, their precise role during the dormancy and sprouting stages of potato growth has not been fully elucidated. Current research provides fragmented insights but lacks a comprehensive synthesis to describe the subtle interactions among auxins in potato physiology throughout the dormancy cycle. As emerging methodologies and new information become available, the understanding of how auxin regulates the transition between dormancy and growth in potatoes is advancing, promising to fill our current knowledge gaps and facilitate more targeted agricultural interventions.

### 4.5. Ethylene

Ethylene (ETH) is a gaseous phytohormone that was first discovered because of its wide range of biological activities. Its exogenous application has been the subject of an increasing number of studies in regulating the sprout development of potato tubers. ETH’s role in affecting dormancy is subtle; it appears to both rely on and influence a complex network of hormonal signals and environmental factors.

ETH generally acts as a sprout growth inhibitor, extending ecodormancy in many storage organs, including potatoes, onions, and sweet potatoes [102,103,104]. ETH treatment enhances metabolism by inducing sucrose-phosphate synthase (SPS), BAM, AI, and NI activity, thereby modulating carbohydrate metabolism and growth [105,106]. In addition to changes in carbohydrate metabolism, ETH affects the phenylpropanoid pathway, reducing total phenols and accelerating their degradation, which contributes to the inhibition of sprouting [107].

Contradictions in ETH’s interaction with dormancy regulation are observed when considering exposure duration. While short-term ETH applications are known to disrupt dormancy, leading to sprouting, prolonged exposure is imperative to sustain dormancy. The mechanism behind this involves an up-regulation of the ABA catabolism-related gene, *StCYP707A2*, and an increase in coumaric acid levels, resulting in reduced ABA levels and facilitating sprouting [106]. Despite this, there exists some controversy within scientific discussions regarding ETH’s interactions with ABA. Correspondingly, the relationship between continuous ETH application and increased ABA levels has also been documented, positioning ABA as a significant factor in maintaining tuber dormancy [108,109]. Genetic analyses reinforce the notion that ETH’s influence extends beyond its own pathway, significantly altering auxin, CK, and brassinosteroid pathways. A notable downregulation of genes associated with plant growth and auxin signal transduction post-ETH treatment suggests sophisticated crosstalk between hormonal pathways [110].

During storage, ethylene production in potato tubers remains low [109], and its role in tuber dormancy is not fully understood. The presence of ethylene-responsive mechanisms suggests complex ecological roles or widespread adaptive traits in plant tissues. However, although ethylene can prolong dormancy, the mechanism by which it inhibits germination remains unclear. There is controversy regarding the effect of ethylene on ABA levels in potatoes, yielding conflicting results. Therefore, it is crucial to consider environmental factors during ethylene treatment, as they may contribute to the observed differences. Studies on ethylene sensitivities and responses among potato cultivars could further elucidate the complexity of how exogenous ethylene affects dormancy. This complexity deserves further exploration for potential agricultural applications.

### 4.6. Brassinosteroids

Brassinosteroids (BRs) are key plant steroids that coordinate multiple physiological processes, including stomatal development, morphogenesis, flowering, vascular tissue formation, and seed germination. The study conducted by Korableva et al. [111] on the influence of BR on apical meristem growth, ETH synthesis, and ABA content in potato tubers underscores a significant finding: the potent BR known as 24-epibrassinolide significantly prolongs deep dormancy while concurrently augmenting ETH production and facilitating the accumulation of both free and bound forms of ABA in potato buds.

Contrastingly, a more recent investigation by Li et al. [112] presented that 500 nM 24-epibrassinolide accelerates potato sprouting. This was associated with significant alterations in the phosphoproteomic profile of the potato after BR treatment, where quantitative analysis highlighted BR’s role in promoting tuber sprouting by modulating phosphorylation patterns of proteins, thereby hastening the conversion of starch to soluble sugars during sprout development [112]. Applying external treatments of BR and GA3 to potato tubers effectively stimulates sprouting and promotes the development of robust, healthy buds [113]. Kim et al. [114] complemented these insights by demonstrating that exogenous BR can inactivate ABA signaling pathways while simultaneously activating downstream genes of GA, revealing an intricate interaction among these growth regulators.

Furthermore, it is noteworthy that ABA suppresses seed germination and post-germinative growth in *Arabidopsis thaliana*. Conversely, BRs counteract this inhibitory effect of ABA, thereby promoting these critical growth stages [115]. The major signaling output of BRs is regulated by ABA signaling. The underlying molecular mechanisms involve the protein kinase Brassinolide Insensitive 2 (BIN2), a critical checkpoint in BR signal transduction, which mitigates BR signaling through the phosphorylation of the transcription factors BES1 and BZR1 [116]. *Arabidopsis* plants overexpressing *BIN2* showcased intensified ABA-mediated suppression of seed germination, underpinning the role of BIN2 in reinforcing ABA signaling, pivoting around the ABI5 signaling factor status [117]. Notably, in potatoes, the StBIN2 protein amplifies ABA signaling by phosphorylating SnRK2.3 [118]. Furthermore, overexpression of *StBIN2* was found to prolong tuber dormancy, with the opposite effect observed when this gene was silenced, resulting in an earlier sprouting period [119]. The interaction between StSN2 and StBIN2 via distinct cysteine residues and their joint contribution to tuber dormancy reveals the delicate landscape of BR and ABA signaling within tuber plant tissues.

Over the past few years, research has incrementally but meaningfully advanced our understanding of how BR affects the initial stages of sprouting in dormant potato tubers. The focus has largely been on the practical effects of the external application of BR on breaking dormancy and promoting sprouting. However, the understanding of how BR production within plants affects these processes remains incomplete. A thorough investigation of BR biosynthesis in potatoes and its regulatory role in dormancy is urgently needed. An in-depth study of these biological pathways goes beyond theoretical interest; it has the potential to improve the consistency of potato tuber sprouting, which is of great importance for seed potato production.

### 4.7. Jasmonates

Jasmonates (JAs) encompass a group of compounds including jasmonic acid, its methyl ester (MeJA), and its isoleucine conjugate (JA-Ile) [120]. The aromatic compound MeJA was originally isolated from the essential oil of *Jasminum grandiflorum* L. [121]. Subsequently, free jasmonic acid was extracted and identified in the culture filtrate of the fungus *Lasiodiplodia theobromae* [122]. This discovery was significant as it provides an initial insight into the physiological role of jasmonic acid, revealing its capacity to inhibit plant growth [123,124]. JAs not only enhance various processing properties but also inhibit sprouting in potato tubers. Dogramaci et al. [125] proposed that treatment with exogenous MeJA effectively suppresses sprouting in ‘Russet Burbank’ potato tubers during long-term storage periods. This treatment leads to reduced enzyme activity related to carbohydrate metabolism in the bud meristem, indicating the downregulation of primary metabolism in these dormant tissues. Furthermore, MeJA exposure induces an upsurge in gene expression linked to the metabolism of hormones like ABA, BR, salicylic acid (SA), and mechanisms of dormancy regulation.

The study of JAs on potato dormancy and sprouting is an intriguing field. However, the current research on the specific effects and mechanisms of JAs on potato dormancy and sprouting is still relatively limited. Future studies can further explore the role of JAs in the process of potato dormancy and sprouting, as well as how to optimize potato cultivation and storage strategies by regulating the levels and activities of JAs.

### 4.8. Strigolactones

Strigolactones (SLs), a novel class of plant hormones, were initially recognized for their role in inducing the germination of parasitic plants like *Striga* and *Orobanche* [126]. SLs are synthesized from carotenoids via an oxidative cleavage process that is facilitated by enzymes known as carotenoid cleavage dioxygenases [127,128,129]. Currently, CCD7 is thought to be responsible for catalyzing the 9,10 cleavage of 9-cis-β-carotene, resulting in the formation of 10′-apo-β-carotenal and β-ionone. Subsequently, CCD8 cleaves the 10′-apo-β-carotenal to yield C18-ketone β-apo-13-carotenone. This intermediate is then rapidly transformed into carlactone by CCD8, through a complex sequence of reactions [130]. Transgenic potato plants (cultivars of ‘Désirée’)—engineered with RNAi to suppress the expression of *CCD8-a*, a crucial gene in the biosynthesis of SLs—showed an increase in secondary growth and a decrease in dormancy, suggesting that SLs exert an inhibitory effect on sprouting [131]. Notably, these *ccd8*-RNAi tubers showed a diminished reaction to gibberellic acid (GA3) in contrast with wild-type tubers when assessed using an in vitro sprout induction assay [131], suggesting an interaction between SLs and GAs, which warrants further detailed investigation. Currently, the research on the specific effects and mechanisms of SLs on potato dormancy and sprouting is still relatively limited.

### 4.9. Salicylic Acid

Salicylic acid (SA) is a plant hormone known for its role in plant growth and development, the stress response, and defense against pathogens [132]. Its functional scope extends to the modulation of dormancy and sprouting in potato tubers. The administration of SA to potatoes can alter the duration of dormancy by interacting with a range of physiological pathways. SA has been reported to extend the dormancy period of potato tubers by reducing starch breakdown and increasing glycoalkaloid levels [133]. This results in delayed sprouting and growth, which is beneficial to potato storage and shelf life. However, the specific mechanism by which salicylic acid regulates potato dormancy and sprouting still lacks in-depth research.

## 5. Summary and Perspective

Potato dormancy, a physiological state in which the tuber remains inactive and unresponsive to sprouting stimuli, has long been a subject of intense research. Scientists around the world have been investigating the underlying mechanisms that regulate this complex biological process. Significant progress has been made in understanding the role of various plant hormones, particularly ethylene and brassinosteroids, and environmental factors such as magnetic fields, cold plasma treatment, and UV-C irradiation in controlling potato dormancy and sprouting. Studies have revealed that hormones can interact with each other and influence potato tuber dormancy and sprout development. Moreover, genetic and transcriptional studies have identified key genes and transcription factors involved in dormancy regulation. These findings have not only enhanced our understanding of the molecular basis of potato dormancy but also provided potential targets for manipulating this trait in crop improvement programs.

However, despite these advancements, many unanswered questions and challenges remain in this field. The precise interactions among different hormones and their receptors, as well as the downstream signaling events, remain incompletely understood. Furthermore, the environmental factors that affect potato dormancy and their interactions with genetic and hormonal factors are also active areas of research.

Moreover, the genetic makeup of the potato and the environmental conditions it is grown in also play important roles in dictating the span of tuber dormancy. Potato cultivars with shorter tuberization tend to have shorter dormancy phases. Conversely, cultivars with longer tuberization periods generally enter a longer dormancy, delaying sprouting and extending the storage potential. Understanding the genetic variations influencing these periods can lead to the manipulation of biochemical pathways, enhancing tuber development and allowing for adjustments in shelf life and crop resilience. The external factors influencing potato mother plants are just as important as the genetic ones. Potatoes cultivated in cooler climates often exhibit extended dormancy periods, while warmer conditions tend to shorten this dormancy. Additionally, agricultural practices, particularly fertilization, significantly influence the duration of dormancy. An increased application of nitrogen fertilizer may accelerate growth, potentially leading to a reduction in the dormancy period. Conversely, reduced levels of nitrogen could result in prolonged dormancy.

Future expectations for potato dormancy research depend on assembling these fragments into a coherent picture and translating this knowledge into viable strategies for agricultural and storage practices. Researchers anticipate that the convergence of genetic insights and biotechnological advancements will allow for the precise control of dormancy periods to meet the needs of different regions and markets. Collaboration between basic research and practical applications is expected to generate innovative, sustainable, and accessible solutions for managing potato dormancy, thus making a significant contribution to the efficiency of the global potato industry.

In summary, potato dormancy research is an active and exciting field with great potential to improve crop performance and sustainability. With continued efforts and advancements in technology, it is expected that we will gain a deeper understanding of dormancy mechanisms and develop more effective strategies to manipulate this trait in potatoes and potentially other crops as well.
